# 

**Published:** 1897-12

**Authors:** 


### MARRIAGES.

HOULDSWORTH—WARD.—On Wednesday evening, October 27th, at the residence of the officiating minister, Rev. Sumner Wynne Stevens, 5513 Hunter's avenue, West Philadelphia, Dr. Joseph D. Houldsworth, of Roxborough, to Miss Hattie Ward, of West Philadelphia.

PAXSON—NOBLET.—On Friday evening, October 28th, at the Cathedral of Sts. Peter and Paul, Chicago, Ill., Dr. Harry Darlington Paxson to Miss Etta Lawrence Noblet.

### PERSONALS.

Drs. W. E. A. Wyman and A. C. Manuel are doing post-graduate work at McKillip Veterinary College.

Dr. Joseph Hughes was called as an expert in the famous Luetgert murder trial in Chicago.

Dr. J. Wagner, of Tavistock, Canada, fills the rôle of Secretary to the Perth Veterinary Association.

Dr. E. M. Ranck, of Philadelphia, has identified himself with the Biological Laboratories of the Messrs. H. K. Mulford Co., as assistant to Dr. John W. Adams.

Dr. F. C. Grenside has entered private practice in New York City, after spending several years as manager of well-known breeding-farms.

Veterinarian G. Howard Davison, of Milbrook, New York, has been elected Secretary of the National Association of Live-stock Exhibitors.

Dr. R. S. Huidekoper has returned to New York City for the winter season.

Dr. E. A. A. Grange, of Detroit, Mich., has identified himself with the laboratories of Messrs. Parke, Davis & Co., engaged in the preparation of antitoxin and other biological products.

Veterinarian J. G. Stewart, of Brantford, Ontario, was recently elected President of the provincial master-shoers' organization.

Dr. Tait Butler fills the post of Secretary for the Mississippi Stock-breeders' Association.

The *Spirit of the Times*, of New York, of November 13th, in its New York Horse-show number, has a photo-reproduction of Dr. William Sheppard, the senior veterinary inspector of the show since 1890.

Dr. C. J. Sihler, United States meat-inspector, of Kansas City, Mo., has resigned his position to enter private practice with Dr. R. C. Moore as associate.

Among the veterinarians in attendance at the session of the American Public Health Protective Association were noted Drs. D. E. Salmon, Leonard Pearson, F. S. Allen, James Johnston, W. Horace Hoskins, C. J. Marshall, John W. Adams, and Frank Standen.

Dr. Joseph R. Jefferis, of Wilmington, Del., has removed to Florida, where he will devote his time and attention to an orange-grove and farming.

Drs. D. E. Salmon and Leonard Pearson presented excellent papers at the American Public Health Association, both of which awakened much interest, and will aid the advance of keener attention to our food-products.

Dr. O. C. Farley, of New York City, filled the place of the late Dr. Edward Loomes as veterinary inspector at the National Horse-show.

Dr. J. I. Gibson, of Denison, Iowa, fills the post of State Veterinarian of that State.

Dr. H. L. Ramacciotti, of Omaha, Neb., holds the position of city veterinarian and food-inspector and live-stock inspector for the Union Pacific Railway.

Dr. Lawrence Campbell, of Chicago, Ill., has retired from the practice of veterinary science, and will shortly enter upon other work that will take him from Chicago.

Drs. D. E. Salmon and A. D. Melvin, of the Bureau of Animal Industry, were visitors to Buffalo in November, investigating charges lodged against Dr. Nelson P. Hinkley, chief inspector of that port. These charges were the outgrowth of the discharge of several subordinates for conduct prejudicial to the service.

---

### PENNSYLVANIA STATE BOARD OF VETERINARY MEDICAL EXAMINERS.

This board will hold their midwinter examination of all applicants for a license to practise in Pennsylvania at 10 A.M. on the third Monday and third Tuesday in December (20th and 21st) on the third floor at the northwest corner of Broad and Market Streets, Philadelphia. For further information address

Dr. S. J. J. Harger,
Secretary.
205 North Twentieth Street, Philadelphia.

W. Horace Hoskins, D.V.S.,
President.

### INDEX.

ABDOMINAL surgery, a few cases of, 711
Abele, Francis, 734
Abortion in domesticated animals, 75, 124, 211
Acetanilid, 10, 84
Acromegaly in a dog, case of, 444
Actual cautery, 692
Actinomycosis, 371
 on the finger (taken from a calf), 225
Acute indigestion in cow, 769
 laminitis, 366
 synovitis, or open joint, 131
Address of welcome of Major John J. McCann at Nashville on behalf of the State and the Tennessee Centennial, 686
 Wisconsin Veterinary Society, 373
Allen, F. S., M.D., D.V.S., 10, 84
Alumni Association (Annual Address of the President of), American V. College, 423
Always read these selections, 648
American Veterinary Medicine, history of, 34
Among the Colleges:
 American Veterinary College, 337
 Chicago Veterinary College, 336
 Columbian University, Veterinary Department, 326, 595
 Indiana Veterinary College, 326
 Kansas City Veterinary College, 409
 McKillip Veterinary College, 325
 New York College of Veterinary Surgeons, 320
 New York State Veterinary College, 462, 594
 Ontario Veterinary College, 327
 United States College of Veterinary Surgeons, 330, 595
 University of Louisiana, 595
 Veterinary Department, Harvard University, 594
 Veterinary Department, University of Pennsylvania, 461
Amos, Walter, V.S., 562
Andropogon sorghum, poisoning cattle, 679
Animals, abortion in domesticated, 211
Animal diseases, communicable, 87
Announcement, important, 230
Antifebrin, 10, 84
Antitoxin, use of tetanus, 496
Antivivisection bill in the U. S. Senate, 504
Antrum, cystic disease of, 773
Appointment well merited, 579
Army legislation, 28
Arrowsmith, W. H. D.V.S., 392
Artificial blood-serum, injection for toxæmia, extensive hemorrhage, 390
Arytenectomy operation by electrical lamp, 392
Atresia of the naris in a mare, 274
Austria, reorganization of veterinary studies, 304
Azoturia, 286, 382, 440

BABB, ALBERT, A.B., M.D.C., 44, 248
Bacteriology in its relation to veterinary science, 12, 79
Baker, A. H., D.V.S., 283
Better safeguards needed, 579
Birds, tuberculosis of, 129
Bitting, A. W., D.V.M., 560
Boards of examiners, 464, 527
Bolley, H. L., M.S., 543
Book reviews, 317, 405, 458, 795
Bowley, C., 353
Braginton, Fred., V.S., 231
Buckley, E., 414, 471
Bulletin No. 64 of the Virginia Agricultural Experiment Station, 581
Bureau of Animal Industry, reports, 581
Burkholder, A. J., D.V.S., 127

CALIFORNIA, control work in, 586
 State V. M. Association, 735
Campbell, A. B., V.S., 15
 Lawrence, 48, 184, 178, 256, 351
Canada, control work in, 42, 351, 416, 468, 593
Canine practice, some salient points in, 562
Cannabis Americana-indica, 199
Cathartics, a study of, 171, 177
Cattle-tick plague, preventive treatment, 67
Chicago Veterinary Society, 49, 114, 177, 254, 350, 414, 467, 592, 734
 resolutions passed by the, 417
 College, commencement exercises, 336
 Association, work in, 99
Clark, B. L., M.D.C., 366
 J. L., V.S., 428
 W. G., 113, 176
Clement, A. W., D.V.S., 135, 271
Cloverdell farm, four interesting cases of, 714
Colic, 482
Colorado veterinarians seek recognition, 29
 proposed bill to regulate practice of veterinary surgery, 35
 State V. M. Association, 593
Colleges, need oj veterinary education in medical, 615
College, Veterinary Society of Harvard, Class of '99, 352
Colt, abnormal condition of forelegs, 637
 intussusception of small intestine, 779
Colts, nitric acid treatment for umbilical hernia in, 143
Commencement exercises:
 American Veterinary College, 337
 Chicago Veterinary College, 336
 Cornell University, 462
 Indiana Veterinary College, 326
 Kansas City Veterinary College, 409
 McKillip Veterinary College, 325
 New York College of Veterinary Surgeons, 320
 Ontario Veterinary College, 59, 327
 United States College of Veterinary Surgeons, 330
 Veterinary Department of Columbian University, 326
 Veterinary Department of Harvard University, 528
 Veterinary Department of the University of Pennsylvania, 461
Communicable animal diseases, 19, 87
Connecticut, 157, 239, 513
Conception and pregnancy in mare, observation on, 121, 206
Contribution to the study of the horse-chestnut, 90, 428

Control work, 786
Belgium, 720
Canada, 42
Connecticut, 239
Illinois, 42
Indiana, 102, 173
Kansas. 516
Maine, 173
Maryland, 41, 647
Massachusetts, 101
Michigan, 174
Minnesota, 583
Mississippi, 518
Missouri, 316
Montana, 239, 517
National, 174, 239, 316, 517
New Hampshire, 518
New York 102
Nevada, 317
Ohio, 173
Ontario, 516
Pennsylvania, 41, 100, 174, 240, 316, 515, 647
South Africa, 42, 720
Texas, 174
Virginia, 240
Correspondence, 168, 237, 313 583
Canada and Manitoba at Nashville, 519
Encouraging the ignorance of rabies, 521
Recent changes in the United States Bureau of Animal Industry, 39
Cow, acute indigestion in, 769
traumatic pericarditis in a, recovery, 451
Coxitis in a mule, 785
Cunningham, R. H., M.D., 444
Currie, J. M., 470
Curtice, Cooper, V.S., 67
Cystic disease of antrum, 773
Cystoma dermalis, 227

Dairy inspection, 231, 430, 745
Dalrymple, W. H., 124, 145, 211, 339, 762
Dawson, Charles F., 75
Delaware, 512
Dell, H. H., 780
J. A., V.S., 121, 206
Dentistry, veterinary, 219
Dermalis cystoma, 227
Detrich, Rev. T. D., 362
Diagnosis and course of moon-blindness in army horses, 493
Diagnostic worth of mallein and tuberculin, 303
Discussion on tuberculosis, 71, 106
Disease and millet, 207
foot and mouth, 702
horse on Kankakee River, 94
Diseases, communicable animal, 19, 87
in man, infectious, 630
of poultry, 34
District of Columbia, 512
Association of the, 350, 591, 673
Dog, case of acromegaly in a, 444
hypertrophy of the prostate in, 144
hysteria, 306
obstruction of intestine, laparotomy, 142
therapeutics of chronic otitis in the, 627
tuberculosis in the, 707
Domesticated animals, abortion in, 75, 124, 211
Dougherty, William, D.V.S., 692
Drift of thought, 66

Editorials, 394
Alaska-Klondike, 577
Always give credit where due, 718
A much-needed organization, 396
Anti-vivisection bill in the U. S. Senate, 504
An excellent appointment, 153
selection, 505
A national misfortune, 27
A peculiar experience, 395
Army legislation, 28

Editorials—
Association work in Virginia and Chicago, 99
A word to the South—a thought for the North, 230
A word as to legislation, 229
A well-merited appointment, 579
Be up and doing, 149, 718
Better safeguards needed, 579
Be considerate; sanitary police work will only grow as we thoughtfully direct it, 151
Closer affiliation demanded, 31
Colorado veterinarians seek state recognition, 29
For good roads, 151
Future meetings of the U. S. V. M. A., 30
of serum-therapy, 577
Good still increases, 504
Go slow, Mr. Secretary, 307
in New York State, 150
Important announcement, 230
Journal—1897—1898, 787
Keystone State, 148
Let the shoemaker stick to his last, 787
Make short work of it, 231
Move right along in this line, 457
Nashville meeting, 641
the Mecca for 1897, 155
New York, legislation in, 98, 154
Not always a wise procedure, 311
On to Nashville, 456
Ontario sets a bad example, 789
Our broadest future is yet before us, 454
secretaries of agriculture, 145
Professor Bang's views, 229
Same thing from different points of view, 718
Should now receive consideration, 580
Something for the consideration of faculties, 394
Stick to it, fellow veterinarians, 394
Sure to have support, 457
Tennessee joins Illinois, 395
The Keystone State's important step, 502
Three more within the fold, 150
To our subscribers, 455
The value of wise sanitary measures, 30
veterinary profession, 576
way of the transgressor is hard, 579
Two excellent selections, 233
U. S. V. M. A. proceedings, 503
Unprofessional conduct in a member of the U. S. V. M. A., 100
Wake up, Connecticut, 231
Well done, Montana, 147
-deserved recognition, 580
What we want to know, 31
Where next? 578
Why is it thus? 506
Will it prove a wise departure? 644
Winnipeg a good example, 502
Ensilage, silos, and the value of, 362
Epidemic of purulent inflammation of the milk-ducts of cows, 135
Equine nasal polypi, with profuse discharge, 631
Establishment of a veterinary institution in Prague, 303
Etiology and treatment of anasarca, 631
Examination for city veterinarian, Philadelphia, 464
of horses for soundness, 127, 299
questions, Ohio State Board of Examiners, 1897, 587
Examiners, Pennsylvania State Board of Veterinary Medical, 184, 737
State boards of veterinary medical, 416
Exercise, value of, 765
Exercises in equine surgery, 582
Experiments in the South, 142
Ellis, Robert W., 108, 177, 261, 411, 469, 732

Extracts from foreign journals:
A new stud farm in Hungary, 493
A report of expulsion of œstrus equi larva (bots) of horse, 223
Arthritis and synovitis, by Dr. Guittard, 632
Cause of Addison's disease, 26
Central association (in Austria) for the erection of public slaughter-houses, 705
Chlorobarium, 91
Contribution to the study of horse-chestnut, 90
Count Gattenberg, committee celebrates twenty-fifth anniversary, 630
Demand for the reorganization of veterinary studies in lower Austria, 304
Diagnosis and course of moon-blindness in army horses, 493
Diagnostic worth of mallein and tuberculin, 303
Equine nasal polypi with profuse discharge, 631
Establishment of a veterinary institution in Prague, 303
Etiology and rational treatment of anasarca, by Dr. Cadeac, 631
Foot and mouth disease, 702
Hypertrophy of the prostate in dogs, 143
Infectious diseases in man, 630
material of the aphtha epidemic, 630
Inoculation of tuberculosis of mammiferæ on gallinaceæ, 22
Inoculations against swine erysipelas (Rothlauf), 491
Intestinal antisepsis by purgation, 23
Iodide of starch, 706
Length of pace in horses, 93
Marking of domestic animals and use of patent marking-tongs, 387
Nitric acid in treatment of umbilical hernia in colts, 143
Obstruction of the intestine and laparotomy in a dog, 142
One means of telling the sex of a pigeon, 142
Pectoral form of septicæmia hemorrhagica, 219
Peculiarly interesting case of indigestion of the stomach, 707
Poisoning of cattle through a licking-stone, salt-poisoning, 24
Propagation of cattle tuberculosis by fecal matter, 90
Professor Hobday's results in median neurectomy, 94
Sand in the stomach of ruminants, 450
Sexual communication between a dog and a hen, 492
Society news, 304
Some statistics in regard to tuberculosis, 449
Spasm of the neck of the uterus, 142
Syncope in dog for five hours, result of chloral and morphine mixture, 23
Therapeutics of chronic otitis in the dog, 627
Treatment of hygromain in the front-knees of cattle, 24
of obstruction of the œsophagus, 706
of pulmonary emphysema in the horse, 632
Tubercle-bacilli in the feces, 22
Tuberculosis in the dog, 707
Tubercular leptomeningitis basilar, 629
synovitis and metastatic inflammation of the joints, 628
Turpentine in colic, 94
Uticaria in the hog, 304

Facts and fancies, 117, 188
Faculties, something for the consideration of, 394
Fecal stasis, 225
matter, propagation of cattle tuberculosis by, 90
Feces, tubercle-bacilli in the, 22
Feeding of millet and millet disease, 207
Femur, outward luxation of the, 97
Fever, tick, 379
Fish, P. A., D.V.S., 619
Field of veterinary science, 551
Floating cartilage in joints, 489
Fœtus in a sheep, retention of, 226
Foot-lameness, 225
nail-wounds of the, 201
Frame, D. P , 594
Future of serum-therapy, 577
meetings of the U.S.V.M.A., 30

Genesee Valley Veterinary Medical Association, 802
Gill, H. D., V.S., 97, 664
Glanders, chronic, in a mule, 716
Glass, Alex., 223, 631, 706
Gleanings, 64, 118, 261, 422, 540, 646
Guide to meat-inspection, 34
Guittard, Dr., 632

Hanson, H. D., D.V.S., 299
Harbaugh, W. H., 532, 793
Hart, John R., V.M.D., 286, 382, 440
Hay, L., 534
Health and wealth of the nation, its relation to the veterinary profession, 576
Helmer, Jacob, 672
History of American veterinary medicine, 34
Hinebauch, T. D., V.S., 207
Hog-cholera, serum-therapy in, 159, 161
diagnosis of, 339
Hog, urticaria of the, 304
Horse-chestnut, contribution to the study of, 90, 428
-disease on the Kankakee River, 94
luxation of the metacarpophalangeal articulation in a, 452
treatment of pulmonary emphysema in the, 632
ulceration of the stomach of a, 716
Horses for soundness, examination of, 127
length of pace in, 93
Horseshoers, relation of, to veterinarians and veterinary colleges, 97
Hoskins, J. Preston, 22, 90, 142, 219, 303, 387, 449, 491, 627, 702
W. Horace, D.V.S., 475, 484, 684
Howard, L. H., D.V.S., 423
Hungary, a new stud farm in, 493
Hunter, S. L., 58
Hysteria—dog, 306
Hypertrophy of the prostate in dogs, 144
Hygromain in the front knees of cattle, 24

Illinois, 42, 159, 241
Veterinary Medical Association, 43, 247
Important announcement, 230
veterinary association meetings, 184
Index, 237
Indiana, 102, 173
Indigestion of stomach, interesting case of, 707
Infectious diseases in man, 630
material of the aphtha epidemic, 630
pneumonia, 495
Inhalation-pneumonia, 619
Injuries, toe-clip, 227
Inoculation experiments on rabbits; rabies in sheep, 271
Inoculations against swine-erysipelas, 491
Inspection, dairy, 231, 430, 745
and milk, 745
guide to meat, 34
Intestinal antisepsis by purgation, 23
obstruction, laparotomy in a dog, 142
perforation by tænia serrata, 780

Intravenous injection of artificial blood-serum for toxæmia and extensive hemorrhage, 390
Intussception of small intestine of colt, 779
Involuntary twitching of the head relieved by trifacial neurotomy, 426
Iodide of potash in schirrous cord, 782
of starch, 706
Iowa, 104
State Veterinary Medical Association, 243

JACOB, M., 733
Joints, floating cartilage in, 489
tubercular synovitis and metastatic inflammation of the, 628

JOPLING, WILLIAM, 247

KANSAS, 515, 516
Kaupp, B. F., D.V.S., 371
Kentucky, 513
Keystone Veterinary Medical Association, 46, 181, 253, 411, 535, 726

LABERGE, C. Z., V.S., 496
Laminitis, acute, 366
Leech, G. Ed., M.D.C., 373
Legislation, army, 28
California, 723
Colorado, 350
Connecticut, 157, 513
Delaware, 512
District of Columbia, 512
Illinois, 159, 241
Indiana, 102
Kansas, 515
Kentucky, 513
Maryland, 158
Massachusetts, 101
Michigan, 515
Montana, 103, 161
New Jersey, 511
New York, 102, 157
North Carolina, 159
Ohio, 159
Pennsylvania, 100, 511
South Dakota, 241
Virginia, 158
Wisconsin, 241
Length of pace in horses, 93
Leptomeningitis basilar, tuberculous, 629
Lewis, Henry, 181, 245
Lights and Shadows, 65
Lipoma causing death, 305
Liver, rupture of the, 635
Louisiana State University, 595
Lockwood, S., 350, 733
Lowe, Wm. Herbert, D.V.S., 19, 87
Luckey, D. F., 379
Lusson, L. O., V.M.D., 198
Luxation of the femur, 97
of the metacarpo-phalangeal articulation in a horse, 452
Lymphangitis, 15

MACGUIRE, R. J., V.S., 218
Maine, 173
Veterinary Medical Association, 109, 179
Mallein and tuberculin, diagnostic worth of, 303
Manitoba Veterinary Association, 260
Mare, observation on conception and pregnancy, 121, 206
Mares, spaying of, 355
Marmorek, 97
Marriages, 100, 156, 234, 397, 581, 794
Martin, W. J., V.S., 131, 489, 635
Maryland, 41, 158
board, 466
Massachusetts, 101
Veterinary Association, 180, 245, 416, 725
Ontario Veterinary Alumni of, 734
Master horseshoers, Pennsylvania Association of, 673
Mayo, N. S., V.S., 223, 225
McCray, W. E., V.S., 637
McGuire, George W., 430
McLean, C. C., 745
Meat-inspection, guide to, 34
Merillat, L. A., V.S., 219
Methyl-violet, or pyoktanin, 484
Michener, J. C., V.S., 365, 714, 765
E. Mayhew, V.M.D., 711, 779
Michigan, 174, 515
State Veterinary Medical Association, 180, 245
Milk-ducts, epidemic of purulent inflammation of the, 135
Milk and dairy inspection, 745
supply, tuberculosis and, 753
Miller, H. P., 225
Minnesota State V. M. Association, 184, 533
Mississippi, 518
Missouri, 316
Valley V. M. Association, 58, 533 731
Montana, 103, 161, 239, 517
Montreal Veterinary Medical Association, 44, 111, 256, 351, 733
Morris, Claude D., 1, 57
Mule, chronic glanders of a, 7J6

NAIL-WOUNDS of the foot, 201, 638
National, 104, 174, 239, 316, 517
Necrosis totalis of the scapula, 95
Need of veterinary education in medical colleges, 615
Nelson, S. B., V.S., 201
Neurotomy, trifacial, 426
Nevada, 316
New Hampshire, 518
Veterinary Medical Association, 49
New inventions, 604, 678
New Jersey, 511
and vicinity, Veterinary Medical Society of the State of, 414, 447
V. M. Association, 349, 733
New publications, reviews of, 33, 237
Bulletin No. 64 of the Virginia Agricultural Experiment Station, 581
Bureau of Animal Industry, twelfth and thirteenth annual reports, 587
Diseases of the Dog and Their Treatment, 33, 237, 405
of Swine, 795
Encyclopædic Dictionary, 319
Exercises in Equine Surgery, 582
Manual of Veterinary Medicine, 408
Report of Tuberculosis Committee of Massachusetts Legislature, 506
Report on Bovine Tuberculosis, Agricultural Experiment Station, University of Minnesota, 458
Text-book of the Comparative Pathology and Therapeutics of Man and the Domestic Animals, 317
New York, 102, 157
County Veterinary Association, 105, 176, 260, 410, 469, 732
State legislation, 98
Board of V. M. Examiners, 154
Veterinary Medical Society, 52, 728
Niagara and Orleans County V. M. Society, 113
Nighbert, J. D., V.S., 95
Niles, E. P., D.V.M., 226, 615
Noack, Otto G., V.S., 530, 638
North Carolina, 159
V. M. Association, 183, 534

OBERST, J. J., M.D.C., 436
Obituary:
Adamson, J. H., 793
Bell, L. T., 310
Doris, John, 236
Farnum, W. D., 506

Obituary—
Flagg, O. H., 155
Gallagher, Michael, 156
Harbaugh, Wm. Heyser, 793
Harms, H. C., 397
Hart, John R., 235
Hummell, A. L., 792
Lancaster, David L., 156
Loomes, Edward, 719
Peralta, José Marie, 33
Rutherford, J. D., 156
Tag, William, 100
Turley, C. A., 156
Walsh, Daniel, 237
Obstruction of the intestine and laparotomy in the dog, 142
Observations on conception and pregnancy in the mare, 121
Ohio, 159, 173
State Board of Examiners, 1897, examination questions, 587
Oneida County V. M. Society, 48, 253, 469
Ontario, 516
Veterinary Alumni of Massachusetts, 734
Association, 109
Western V. M. Association, 108
Open joint, 131, 283
Operation of arytenectomy, assisted by electrical lamp, 392
Oregon, 104
Outside world, 742
Ovariotomy, 138

PARKER, JOHN M., D.V.S., 607, 693
Parturient apoplexy, four cases of, 637
Pathological shoeing, 567
Pearson, Leonard, B.Sc., V.M.D., 193, 278, 349, 551
R. A., B.S., 189, 292
Pease, Vet. Capt. H. T., F.C.S., F.R.M.S., 679
Pennsylvania, 41, 100, 103, 174, 240, 316, 511, 515
State Board of Veterinary Medical Examiners, 184, 465, 737
V. M. Association, 113, 248, 343, 534, 673
Pericarditis in a cow, traumatic, recovery, 451
Perley, H.S., V.S., 637
Personal, 60, 115, 185, 265, 341, 421, 461, 471, 601, 675, 737, 803
Peters, A T., V.S., 159, 161
Petty, J. W., D.V.S., 184, 495
Physiological variations, 192
Plague, cattle-tick, 67
Plaskett, Joseph, 726
Pleasure of being a veterinarian, 365
Pneumonia, infectious, 495
-inhalation, 619
Potash iodide in schirrous chord, 782
Poultry, diseases of, 34
Poisoning of cattle by sorghum, 679
Presentation of the subject of State control of tuberculosis, 607, 693
Preventive treatment, cattle-tick plague, 67
Proceedings of the U. S. V. M. A., 503
Professor Bang's views, 193, 229
Pure milk, 189, 292
Pulmonary emphysema in the horse, treatment of, 632
Purgation, intestinal antisepsis by, 23
Pyoktanin, or methyl-violet, 484

RABIES in sheep, with inoculation experiments, 271
Ravenel, M. P., M.D., 12, 79, 753
Reactions, interesting tuberculin, 771
Relations of horseshoers to veterinarians and veterinary colleges, 97
Relationship existing between the veterinary profession and the humane societies, 762
Report on the relations of the veterinary profession to the Department of Police of the City of Chicago, 468
Reports of cases, 390
An abnormal condition of the forelegs of a colt, 637
Actinomycosis on the finger, taken from a calf, 225
Chronic glanders of mules, 716
Coxitis in a mule, 785
Cystoma dermalis, 227
Diabetic symptoms from contaminated drinking-water, 495
Foot lameness, fecal stasis, 225
Four cases of parturient apoplexy, 637
interesting cases at Cloverdell Stock Farm, 714
Hysteria dog, 306
Infectious pneumonia, 495
Intestinal perforation by tænia serrata, 780
Intravenous injection of artificial blood-serum for the relief of toxæmia and extensive hemorrhage, 390
Intussception of small intestine of colt, 779
Lipoma, causing death, 305
Luxation of the metacarpo-phalangeal articulation in the horse, 452
Necrosis totalis of the scapula, 95
Operation of arytenectomy assisted by the electrical lamp, 392
Outward luxation of the femur, 97
Post-mortem revealing two spleens, 452
Potash iodide in schirrous chord, 782
Resection of necrotic parts of the soft tissues of the frog for nail-wound, 638
Record of cases of abdominal surgery, 711
Retention of fœtus in a sheep, 226
Rupture of the liver, 635
Spasmodic contraction, with hypertrophy of retractor muscles of the penis of a bull, 783
Tenotomy, 638
Tetanus, the use of antitoxin, 496
Traumatic pericarditis in a cow, 451
Treatment of anasarca by injection of antistreptococcus serum, 97
Ulceration of the stomach of the horse, 716
Resolutions passed by the Chicago Veterinary Society, 417
Reynolds, M. H., M.D., V.M., 171, 177
Rhoads, W. L., D.V.S., 201, 414, 536, 728
Richardson, James M., 452
Ridge, W. H., V.M.D., 769
Robertson, James, 469
Rochester, N. Y., organization of a new veterinary society in, 792
Roub, J. F., D.V.S, 305
Rupture of the liver, 635

SALT-POISONING, 24
Sand in the stomach of ruminants, 450
Sanitarians as veterinarians, 1
Schaffter, E. P., V.S., 482
Schirrous chord, potash iodide in, 782
Schoenleber, F. S., 771
Schuylkill Valley V. M. Association, 470
Science, review of veterinary, 551
veterinary bacteriology in its relation to, 79
Seen and heard in many places, 269
Selections, 339, 573, 633, 708, 777
Serum-therapy in hog-cholera, 159, 161
the future of, 577
Serum diagnosis of hog-cholera, 339
Sheep, retention of fœtus in a, 226
St. Louis veterinarians organize, 416
Significance of the caption of a paper, 560
Silos and the value of ensilage, 362
Simpson, Thomas V., V.S., 138, 390
Smith H. W, V.S., 306
Snively, J. R., V.S., 225

Society proceedings:
California State V. M. Association, 735
Chicago Veterinary Society 49, 114, 177, 254, 350, 414, 467, 592, 734
Colorado State V. M. Association, 593
Illinois State V. M. Association, 43, 247
Iowa State V, M. Association, 243
Keystone V. M. Association, 46, 181, 256, 411, 535, 726, 796
Maine V. M. Association, 109, 179
Manitoba V. M. Association, 260
Massachusetts V. M. Association, 180, 245, 416, 725, 801
Michigan State V. M. Association, 180, 245
Minnesota State V. M. Association, 184, 533
Missouri Valley V. M. Association, 58, 533, 731
Montreal V. M. Association, 44, 111, 256, 351, 733, 798
New Hampshire V. M. Association, 49
New York State V. M. Society, 52, 728
Niagara and Orleans County V. M. Society, 113
North Carolina V. M. Association, 183, 534, 802
Oneida County V. M. Association, 48, 253, 469
Ontario Veterinary Association, 109, 734
Organization of St. Louis Veterinarians, 416
Pennsylvania State V. M. Association, 113, 248, 343, 543, 664
Association of Master Horsheshoers, 673
Board of Veterinary Medical Examiners, 184, 416, 737
Schuylkill Valley V. M. Association, 470
Tennessee, V. M. Association, 183, 725
United States V. M. Association, 184, 243, 354, 418, 466, 651
Veterinary Association of the District of Columbia, 350, 591, 673
Veterinary Medical Association of New Jersey, 349, 414, 471, 733
Veterinary Medical Society of the Ontario Veterinary College, 798
Veterinary Medical Society of the University of Pennsylvania, 113, 176, 259, 353, 732
Veterinary Medical Society of Harvard College, Class of '99, 352
Veterinary Medical Association of New York County, 105, 176, 260, 410, 469, 732, 797
Virginia State V. M. Association, 112
Western Ontario V. M. Association, 108
Wisconsin Society of Veterinary Graduates, 113, 175
Some aspects of association aims and work, 475
experiences in the South, 142–145
Something for the consideration of faculties, 394
to interest each reader, 418
Sorby, Harold, 97
Soundness, examination of horses for, 127
South Dakota, 241
Africa, 42
Spaying of mares, 355
Spindler, John E., 114, 179, 260, 354
Spleens, two, post-mortem, 452
State examining boards, uniformity of State regulations as to, 684
control of tuberculosis, subject of, 607
Boards V. M. Examiners, 416
Stock foods, some poisonous, 223, 225
Stokes, William Royal, M.D., 133, 271
Study of the diseases of poultry, 34
Sunstroke, 198
Sweetapple, C. H., 111
Sweeny, Thomas M., 113, 530
Synovits, acute, 131

TÆNIA serrata, intestinal perforation by, 780
Tennessee Centennial, address of welcome, 689
V. M. Association, 183, 725
Tenotomy, 638
Tetanus. 278, 495, 496
Texas, 174
Things and conditions are ever changing, 539
Tick-fever, 379
Toe-clip injuries, 227
Toxæmia and extensive hemorrhage, intravenous injection of artificial blood-serum for the relief of, 390
Transactions of the Thirty-fourth Annual Meeting of the U. S. V. M. A , Buffalo, N. Y., September, 1896, Nos. 1 to 6.
Cathartics, a study of, 171
Feeding of millet and millet disease, 207
Gill, H. D., the relations of horseshoers to veterinarians and veterinary colleges, 97
Hog-cholera, serum-therapy in, 157
Nail-wounds, of the foot,, 201
Physiological variations, 192
Some experiences in the South, 142
poisonous stock foods, 223
Veterinary dentistry, 219
Traumatic pericarditis in a cow, recovery, 451
Treacy, M. J., 782
Treatment of anasarca by the subcutaneous injection of antistreptococcus serum, 97
Tuberculosis, 71, 436
birds, 129
dog, 707
discussion on, 106
leptomeningitis, basilar, 629
milk supply, and presentation of the subject of State control of, 607, 693
propagation of cattle, by fecal matter, 90
statistics in regard to, 449, 771
tuberculin reactions, interesting, 771
Turner, J. P., 350, 591, 674
Typhoid serum diagnosis, 543

ULCERATION of the stomach of a horse, and chronic glanders of a mule, 716
Uniformity of State regulations as to State examining boards, 684
U. S. V. M. A., 184, 243, 354, 418, 503, 651, 675
future meetings of, 30
on to Nashville, 498
United States experiment veterinarians, 674
University of Pennsylvania, Veterinary Medical Society of the, 113, 178, 259, 353, 732

VALUE of exercise, 765
Variations, physiological, 193
Veterinarians as sanitarians, 1
Veterinary blue-book of New York, 33
Virginia, 158, 240
Agricultural Experiment Station, Bulletin No. 64 of, 581
State board, 465
V. M. Association, 99, 112, 528

WALLIS, W. B., 134
Waugh, James A., V.S., 227
Wagner, Jacob, 108
Western Ontario V. M. Association, 108
West, W. L., 109, 179
White, D S., D.V.M., 783
Why? 201
Williams, W. L., V.S., 192, 193, 274, 355, 426, 451, 452, 619
Charles, V.M.D,, 199
Willyoung, L. E., D.V.S., 227
Wisconsin, 241
Society of Veterinary Graduates, 113, 175, 373
Wyman, W. E. A., V.S., 97, 716

YINGST, W. H., V.S., 567

